# Lymphatic Mapping with Multi-Lymphosome Indocyanine Green Lymphography in Legs with Lymphedema

**DOI:** 10.1055/a-2375-8153

**Published:** 2024-09-17

**Authors:** Hisako Hara, Makoto Mihara

**Affiliations:** 1 Department of Lymphatic and Reconstructive Surgery, JR Tokyo General Hospital, Tokyo, Japan; 2 Department of Lymphatic Surgery, Lymphedema Clinic, Tokyo, Japan

**Keywords:** lymphedema, lymphosome, ICG, lymphatic mapping, indocyanine green

## Abstract

It is observed that the locations of the most functional lymphatic vessels in the lymphedematous limbs can differ significantly from those in healthy limbs. The aim of this study was to elucidate the lymphatic map of lymphedematous limbs. We retrospectively analyzed 59 patients (118 limbs) with lower limb lymphedema. Fifty-five were women and four were men. The mean age and duration of lymphedema was 62.4 and 7.7 years, respectively. For the lateral thigh lymphosome, we injected indocyanine green (ICG) at the lateral knee and measured the distance (Dt) between the anterior superior iliac spine (ASIS) and the point where the lymphatic vessels crossed the reference line (the line connecting the ASIS and the patellar center). For the lateral calf lymphosome, we injected ICG at the lateral ankle and measured the distance (Dc) between the inferior patellar border and the point where the lymphatic vessels crossed the reference line (the anterior border of the tibia). In the lateral thigh, the mean Dt was 30.4 ± 0.6 cm (range, 0–41 cm) and the distribution peaked at approximately 30 cm from the ASIS. In the calf, the mean Dc was 13.1 ± 0.9 cm (range, −11 to 32 cm). The distribution of lymphatic vessel locations was highly variable. We could establish the lymphatic map in the lymphedematous legs. The distribution of lymphatic vessels in the thigh and lower legs had one and two peaks, respectively.

Lymphaticovenous anastomosis (LVA) is a minimally invasive surgical method that can be performed with a small incision under local anesthesia to treat lymphedema. 1 2 3 4 5 6 Among several examinations including lymphoscintigraphy, 7 indocyanine green (ICG) lymphography, 8 9 lymphatic ultrasonography, 10 11 12 13 14 15 16 17 18 or lymphatic magnetic resonance imaging, 19 20 the most common examination performed as a preoperative examination for LVA is ICG lymphography. Conventionally, ICG was injected only at the distal points of the affected limbs (e.g., interdigital web spaces, toe web spaces, or ankles). 8 To observe more lymphatics, we proposed multi-lymphosome ICG lymphography, in which we injected ICG at the dorsum of the foot, the lateral ankle, and the lateral knee, and named the lymphatic vessels in each lymphosome as the saphenous, lateral calf, and lateral thigh lymphatics, respectively. 21 22 23 24 25

In the current study, we performed multi-lymphosome ICG lymphography in lymphedematous patients and attempted to establish a lymphatic map. This map will contribute not only to surgeons but also to therapists who perform manual lymph drainage. This study aimed to elucidate the lymphatic map of lymphedematous limbs.

We retrospectively analyzed 59 patients (118 limbs) who underwent LVA for lower limb lymphedema under local anesthesia between September 2020 and June 2021. Among the participants, 55 were women and 4 were men ( Supplementary Table S1 [available in the online version only]). The mean age and duration of lymphedema were 62.4 years (range, 21–87 years) and 7.7 years (range, 1–34 years), respectively.

In multi-lymphosome ICG lymphography, we injected ICG (0.5% Diagnogreen, Daiichi Pharmaceutical, Tokyo, Japan) at three lymphosomes subdermally and observed the lymphatic pathway with the Photodynamic Eye camera (Hamamatsu Photonics, Hamamatsu, Japan) immediately after injection. 19 20 21 We recorded the location of the lateral calf and lateral thigh lymphatics only, because the route of the saphenous lymphatics is always at the medial leg.

For the lateral thigh lymphosome, we injected 0.05 mL of ICG at the mid-lateral thigh at the superior patellar border. We connected the anterior superior iliac spine (ASIS) and the patellar center to form the reference line. We measured the distance (Dt) between the ASIS and the point where the lymphatic vessels crossed the reference line ( Fig. 1A ).

**Fig. 1:**
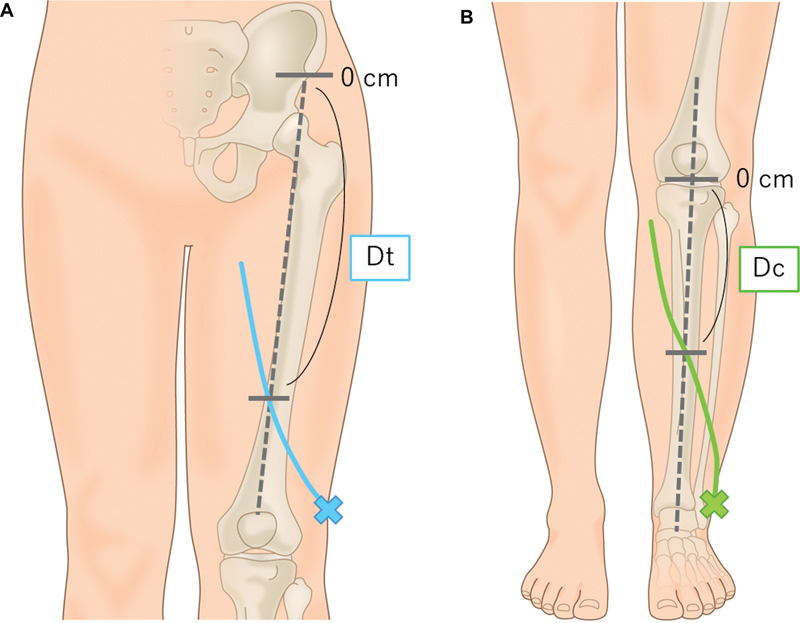
Schematic images of the lymphatic mapping. ( **A** ) For the lateral thigh lymphosome, we injected indocyanine green (ICG) at the mid-lateral thigh at the superior patellar border (blue cross). The reference line connected the anterior superior iliac spine (ASIS) and the patellar center (gray dotted line). We measured the distance (Dt) between the ASIS and the point where the lymphatic vessels crossed the reference line. ( **B** ) For the lateral calf lymphosome, we injected ICG at the superior border of the lateral malleolus (green cross). The reference line was defined as the anterior border of the tibia and its extension (gray dotted line). We measured the distance (Dc) between the inferior border of the patella and the point where the lymphatic vessels cross the reference line. If the lymphatic vessels crossed the reference line at the proximal point from the inferior border of the patella, Dc was indicated by a negative number.

For the lateral calf lymphosome, we injected 0.05 mL of ICG at the superior border of the lateral malleolus. We defined the anterior border of the tibial bone and its extension as the reference line. We measured the distance (Dc) between the inferior patellar border and the point where the lymphatic vessels crossed the reference line ( Fig. 1B ). If the lymphatic vessels crossed the reference line at the proximal point from the inferior patellar border, the Dc value was negative.

If pleural lymphatic vessels were observed in one lymphosome, we recorded all the vessels. If no lymphatic vessels crossed the reference line, we recorded “not applicable (N/A).” If the lymphatic vessels ran from the ankle to the posterior side of the calf, we recorded a “posterior pattern.”

In the lateral thigh lymphosome, we observed 78 lymphatic vessels in 72 limbs. Forty-six limbs had “N/A” results. The mean Dt was 30.4 ± 0.6 cm (range, 0–41 cm). The distribution of lymphatic vessels is shown in Fig. 2 (above). The distribution peaked at approximately 28 to 34 cm from the ASIS. Therefore, the lymphatic vessels are likely located on the lateral side of the reference line in the area distal to this point. In contrast, lymphatic vessels in the area within 22 cm from the ASIS are seldom located at the lateral side of the reference line.

**Fig. 2:**
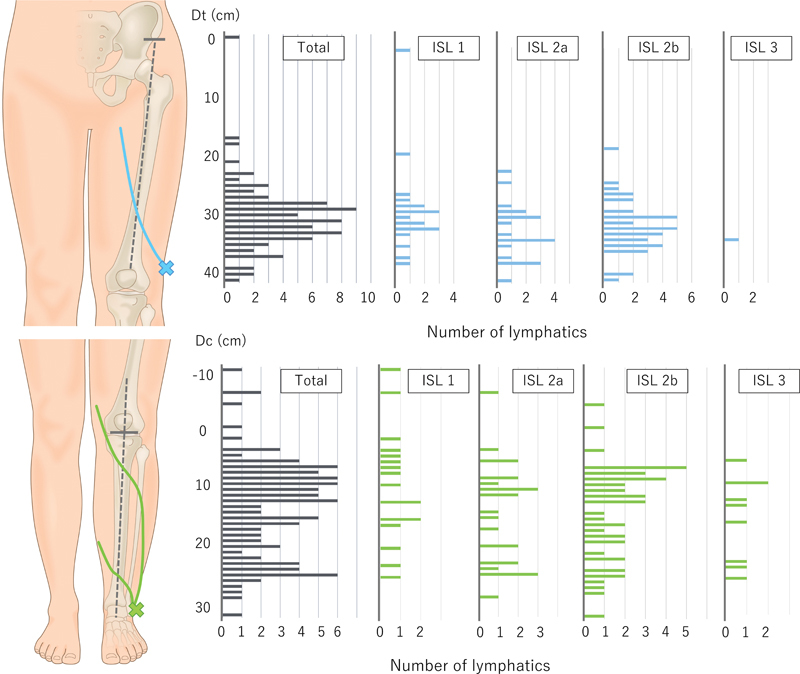
The distribution of the lymphatic vessels. Above: The distribution had a peak at around 30 cm from the anterior superior iliac spine (ASIS). In the area 20 cm proximal to the ASIS, the lymphatic vessels seldom crossed the reference line. Below: The distribution of lymphatic vessel locations was highly variable. In the lower legs, the lymphatic vessels ran laterally in a wide range, from the ankle to the knee joint. ISL, International Society of Lymphology.

In the calf, 107 lymphatic vessels were observed in 88 limbs. We recorded “N/A” and “posterior pattern” in 30 and 9 limbs, respectively. The mean Dc was 13.1 ± 0.9 cm (range, −11 to 32 cm). The distribution of lymphatic vessels is shown in Fig. 2 (below). The distribution of lymphatic vessel locations was highly variable. In the lower legs, the lymphatic vessels ran on the lateral side over a wide range from the ankle to the knee joint.

The body mass indices (BMIs) of those with lymphatic vessels observed and those with “N/A” results were 22.5 and 23.1 kg/m ^2^ , respectively, in the thigh, with no significant difference by Student's *t* -test ( *p* = 0.409). Also, the BMIs were 22.5 and 23.6 kg/m ^2^ , respectively, in the calf, with no significant difference ( *p* = 0.192).

One of the limitations of this study was that we evaluated only the lymphatic vessels that originated from two points on the legs (the lateral ankle and the lateral knee). In reality, there may have been more lymphatic vessels, which should be a topic for future research.

We established a lymphatic map to indicate the variation in where the lymphatic vessels may be located in the lymphedema-affected limb. We hope that the results of the current study will help health care providers involved in the treatment of lymphedema.

## References

[1] Koshima I Inagawa K Urushibara K Moriguchi T Supermicrosurgical lymphaticovenular anastomosis for the treatment of lymphedema in the upper extremities J Reconstr Microsurg 2000 16 06 437 442 10993089 10.1055/s-2006-947150

[2] Hara H Mihara M Ohtsu H Narushima M Iida T Koshima I Indication of lymphaticovenous anastomosis for lower limb primary lymphedema Plast Reconstr Surg 2015 136 04 883 893 26086382 10.1097/PRS.0000000000001631

[3] Chan V S Narushima M Hara H Local anesthesia for lymphaticovenular anastomosis Ann Plast Surg 2014 72 02 180 183 23542832 10.1097/SAP.0b013e31825b3d1e

[4] Mihara M Hara H Tange S Multisite lymphaticovenular bypass using supermicrosurgery technique for lymphedema management in lower lymphedema cases Plast Reconstr Surg 2016 138 01 262 272 27348659 10.1097/PRS.0000000000002254

[5] Mihara M Hara H Furniss D Lymphaticovenular anastomosis to prevent cellulitis associated with lymphoedema Br J Surg 2014 101 11 1391 1396 25116167 10.1002/bjs.9588

[6] Jørgensen M G Toyserkani N M Sørensen J A The effect of prophylactic lymphovenous anastomosis and shunts for preventing cancer-related lymphedema: a systematic review and meta-analysis Microsurgery 2018 38 05 576 585 28370317 10.1002/micr.30180

[7] Maegawa J Mikami T Yamamoto Y Satake T Kobayashi S Types of lymphoscintigraphy and indications for lymphaticovenous anastomosis Microsurgery 2010 30 06 437 442 20878726 10.1002/micr.20772

[8] Yamamoto T Narushima M Doi K Characteristic indocyanine green lymphography findings in lower extremity lymphedema: the generation of a novel lymphedema severity staging system using dermal backflow patterns Plast Reconstr Surg 2011 127 05 1979 1986 21532424 10.1097/PRS.0b013e31820cf5df

[9] Mihara M Hara H Araki J Indocyanine green (ICG) lymphography is superior to lymphoscintigraphy for diagnostic imaging of early lymphedema of the upper limbs PLoS ONE 2012 7 06 e38182 22675520 10.1371/journal.pone.0038182 PMC3366958

[10] Mihara M Hara H Kawakami Y Ultrasonography for classifying lymphatic sclerosis types and deciding optimal sites for lymphatic-venous anastomosis in patients with lymphoedema J Plast Reconstr Aesthet Surg 2018 71 09 1274 1281 30173714 10.1016/j.bjps.2018.05.012

[11] Hara H Mihara M Usefulness of preoperative echography for detection of lymphatic vessels for lymphaticovenous anastomosis SAGE Open Med Case Rep 2017 5 2.050313E6 × 17745207 10.1177/2050313X17745207 PMC5724635 29242747

[12] Czedik-Eysenberg M Steinbacher J Obermayer B Exclusive use of ultrasound for locating optimal LVA sites: A descriptive data analysis J Surg Oncol 2020 121 01 51 56 31612513 10.1002/jso.25728

[13] Hara H Ichinose M Shimomura F Kawahara M Mihara M Lymphatic mapping for LVA with noncontrast lymphatic ultrasound: how we do it Plast Reconstr Surg Glob Open 2024 12 04 e5739 38623448 10.1097/GOX.0000000000005739 PMC11018192

[14] Hara H Mihara M Comparison of various kind of probes for lymphedematous limbs Plast Reconstr Surg Glob Open 2021 9 03 e3490 33968554 10.1097/GOX.0000000000003490 PMC8099413

[15] Hara H Mihara M Diagnosis of lymphatic dysfunction by evaluation of lymphatic degeneration with lymphatic ultrasound Lymphat Res Biol 2021 19 04 334 339 33471593 10.1089/lrb.2019.0071

[16] Hayashi A Giacalone G Yamamoto T Ultra high-frequency ultrasonographic imaging with 70 MHz scanner for visualization of the lymphatic vessels Plast Reconstr Surg Glob Open 2019 7 01 e2086 30859043 10.1097/GOX.0000000000002086 PMC6382250

[17] Bianchi A Visconti G Hayashi A Santoro A Longo V Salgarello M Ultra-high frequency ultrasound imaging of lymphatic channels correlates with their histological features: a step forward in lymphatic surgery J Plast Reconstr Aesthet Surg 2020 73 09 1622 1629 32591265 10.1016/j.bjps.2020.05.053

[18] Visconti G Yamamoto T Hayashi N Hayashi A Ultrasound-assisted lymphaticovenular anastomosis for the treatment of peripheral lymphedema Plast Reconstr Surg 2017 139 06 1380e 1381e 10.1097/PRS.0000000000003362 28406824

[19] Mazzei M A Gentili F Mazzei F G High-resolution MR lymphangiography for planning lymphaticovenous anastomosis treatment: a single-centre experience Radiol Med 2017 122 12 918 927 28770484 10.1007/s11547-017-0795-x

[20] Liu N F Yan Z X Wu X F Luo Y Magnetic resonance lymphography demonstrates spontaneous lymphatic disruption and regeneration in obstructive lymphedema Lymphology 2013 46 02 56 63 24354104

[21] Hara H Mihara M Lymphaticovenous anastomosis for advanced-stage lower limb lymphedema Microsurgery 2021 41 02 140 145 33421191 10.1002/micr.30689

[22] Hara H Mihara M Multilymphosome injection indocyanine green lymphography can detect more lymphatic vessels than lymphoscintigraphy in lymphedematous limbs J Plast Reconstr Aesthet Surg 2020 73 1025 1030 32115379 10.1016/j.bjps.2020.01.021

[23] Hara H Mihara M Multi-area lymphaticovenous anastomosis with multi-lymphosome injection in indocyanine green lymphography: a prospective study Microsurgery 2019 39 02 167 173 30508302 10.1002/micr.30398

[24] Hara H Mihara M Classification of the lymphatic pathways in each lymphosome based on multi-lymphosome indocyanine green lymphography: saphenous, calf, and thigh (SCaT) classification J Plast Reconstr Aesthet Surg 2021 74 11 2941 2946 34024739 10.1016/j.bjps.2021.03.078

[25] Suami H Shin D Chang D W Mapping of lymphosomes in the canine forelimb: comparative anatomy between canines and humans Plast Reconstr Surg 2012 129 03 612 620 22373968 10.1097/PRS.0b013e3182402c6d

