# Supplementary material for: Lymphatic Mapping with Multi-Lymphosome Indocyanine Green Lymphography in Legs with Lymphedema
Source: Arch Plast Surg. 2024 Sep 17;51(6):592–6. doi: 10.1055/a-2375-8153 (PMC11560325; doi:10.1055/a-2375-8153)
Supplement: Supplementary file 1 — Supplementary Material [file 10-1055-a-2375-8153-s24apr0056com.pdf]

Supplementary Table S1 Characteristics of the patients

Number of patients (number of limbs)		59 (118)
Age (years)	Average (range)	62.4 (21–87)
Sex (%)	Female	55 (93.2%)
	Male	4 (6.8%)
Lymphedema duration (years)	Average (range)	7.7 (1–34)
Causative disease (%)	Uterine cervical cancer	31 (52.5%)
	Uterine body cancer	12 (20.3%)
	Ovarian cancer	10 (16.9%)
	Other cancer	3 (5.1%)
	Primary	3 (5.1%)
Lymphadenectomy (%)	+	54 (91.5%)
	−	5 (8.5%)
Radiotherapy (%)	+	32 (54.2%)
	−	27 (45.8%)
ISL classification (limbs; %)	1	27 (22.9%)
	2a	24 (20.3%)
	2b	54 (45.8%)
	3	13 (11.0%)
Lymphoscintigraphy stage (limbs; %)	1	26 (22.0%)
	2	20 (16.9%)
	3	40 (33.9%)
	4	20 (16.9%)
	5	12 (10.2%)

Abbreviation: ISL, International Society of Lymphology.

 © 2024. The Author(s).
